# Infertility in Men: Advances towards a Comprehensive and Integrative Strategy for Precision Theranostics

**DOI:** 10.3390/cells11101711

**Published:** 2022-05-22

**Authors:** Mourad Assidi

**Affiliations:** 1 Center of Excellence in Genomic Medicine Research, King Abdulaziz University, P.O. Box 80216, Jeddah 21589, Saudi Arabia; mourad.assidi@gmail.com; Tel.: +966-(012)-6402000 (ext. 69267); 2 Medical Laboratory Department, Faculty of Applied Medical Sciences, King Abdulaziz University, P.O. Box 80216, Jeddah 21589, Saudi Arabia

**Keywords:** male infertility, sperm, etiology, aging, biomarkers, lifestyle, multiomics, precision theranostics

## Abstract

Male infertility is an increasing and serious medical concern, though the mechanism remains poorly understood. Impaired male reproductive function affects approximately half of infertile couples worldwide. Multiple factors related to the environment, genetics, age, and comorbidities have been associated with impaired sperm function. Present-day clinicians rely primarily on standard semen analysis to diagnose male reproductive potential and develop treatment strategies. To address sperm quality assessment bias and enhance analysis accuracy, the World Health Organization (WHO) has recommended standardized sperm testing; however, conventional diagnostic and therapeutic options for male infertility, including physical examination and semen standard analysis, remain ineffective in relieving the associated social burden. Instead, assisted reproductive techniques are becoming the primary therapeutic approach. In the post-genomic era, multiomics technologies that deeply interrogate the genome, transcriptome, proteome, and/or the epigenome, even at single-cell level, besides the breakthroughs in robotic surgery, stem cell therapy, and big data, offer promises towards solving semen quality deterioration and male factor infertility. This review highlights the complex etiology of male infertility, especially the roles of lifestyle and environmental factors, and discusses advanced technologies/methodologies used in characterizing its pathophysiology. A comprehensive combination of these innovative approaches in a global and multi-centric setting and fulfilling the suitable ethical consent could ensure optimal reproductive and developmental outcomes. These combinatorial approaches should allow for the development of diagnostic markers, molecular stratification classes, and personalized treatment strategies. Since lifestyle choices and environmental factors influence male fertility, their integration in any comprehensive approach is required for safe, proactive, cost-effective, and noninvasive precision male infertility theranostics that are affordable, accessible, and facilitate couples realizing their procreation dream.

## 1. Introduction

Infertility has been defined as a reproductive disease that prevents a healthy woman from conceiving after at least 12 months of regular unprotected sexual intercourse [1]. Male infertility encompasses any health issue that impedes the likelihood of conception and can be caused by abnormal sperm function or obstructions that prevent ejaculation. Multiple factors, including illness, injury, chronic morbidity, and lifestyle choices, contribute to its onset and progression [2]. Male fertility is largely determined in spermatogenesis, the development of spermatozoa from spermatogonia in the testes. This meticulous developmental process is marked by both mitotic and meiotic divisions, followed by extensive morphological and biochemical differentiation, leading to a mature spermatozoan. Male infertility is attributed to abnormal spermatozoa parameters (spermatogenic failure), such as total absence (azoospermia), low count (oligozoospermia), abnormal morphology (teratozoospermia), and/or abnormal motility (asthenozoospermia).

Globally, infertility affects 15% of couples at reproductive age, with male infertility accounting for up to half of all cases [2,3]. The age-standardized prevalence of male infertility reportedly increases by 0.3% annually [4]. However, the increased male factor infertility rate was geographically inconsistent and ranged from 20–70% [4].

Male infertility rates may be underestimated because of cultural differences, social dilemmas, and patriarchy preventing accurate sampling and analysis [4]. In men, it can also trigger anxiety about the stigma of hegemonic masculinity. It is particularly challenging in pronatalist societies, where both virility and fertility are considered hallmarks of manhood, but also in Western societies, where male infertility and impotence are conflated. Paradoxically, assisted reproduction technologies (ARTs) can create additional layers of stigma and secrecy [4]. Moreover, male infertility is associated with significant psychosocial and marital stress, increased cancer risk, poorer overall health, and decreased life expectancy [4].

Semen quality, especially sperm concentration and motility, is the most widely accepted diagnostic marker of male infertility. The WHO has stipulated standard operating procedures (SOPs) for sperm parameters’ analysis [5] to prevent assessment bias and enhance analysis accuracy. However, certain limitations, including ambiguous threshold values, affect the reliability of semen analysis. Irrespective of the ongoing effort to refine these reference values into more relevant subcategories, such as subfertile, indeterminate, and fertile groups, the standard approaches still lack accuracy, reproducibility, and therapeutic efficacy.

## 2. Male Infertility: Complex and Interconnected Roots

**Figure 1:**
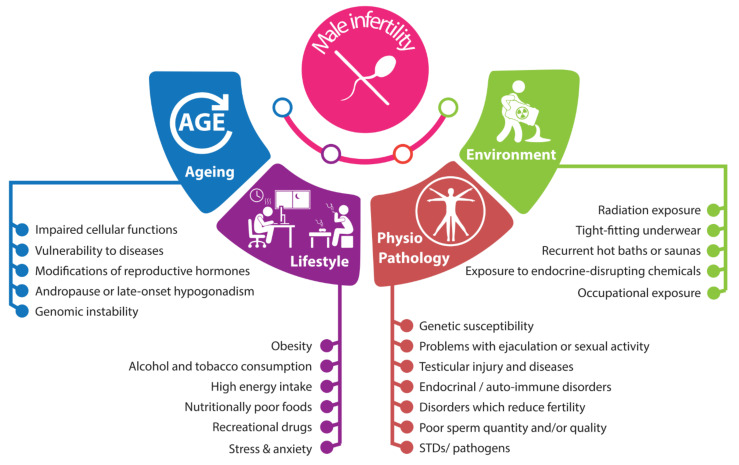
Multifactorial etiology of male infertility.

The lack of progress in treating male infertility owes largely to the underlying complex etiology resulting from interactions among genetics, lifestyle choices, environmental factors, and comorbidities (Figure 1). The influence of aging on male fertility has also become more pronounced with the modern trend of planning pregnancy at later ages (≥40 years) [6].

### 2.1. Anatomo-Pathophysiological Factors

Several anatomical and pathophysiological factors influence male infertility, including blockage of sperm ducts (epididymis or vas deferens), ejaculation complications, testicular injury/disease, hormonal disturbance, genetic disorders, and other medical conditions (e.g., iatrogenic factors). Duct obstruction, which prevents ejaculation, occurs in ~5% of infertile men and is indicative of azoospermia or severe oligozoospermia [7]. Testicular injury/disorder can cause hormonal imbalance, sexual dysfunction, and infertility [8]. Retrograde ejaculation, a dysfunction of the bladder sphincter, is manifested by semen ejaculation into the bladder and causes ~2% of infertility cases [9].

Male reproductive hormones are essential for sexual development and function. Some endocrine disorders related to the hypothalamus, pituitary, and testicular glands can cause infertility through the malfunctioning of sexual hormones and/or compromised sperm production [10]. Sperm antibodies released in certain autoimmune disorders also impair sperm function. Varicocele, an excessive enlargement of the scrotal veins, results in retrograde blood flow and may cause infertility [9].

Besides certain sexually transmitted diseases (STDs) that infect the reproductive tract and induce infertility [11], some bacteria [12] and the Zika virus [13] induce infertility. Indeed, several pathologies of the male reproductive system, such as genitourinary tract infection, induce oxidative stress (OS), associated with male infertility [14,15].

### 2.2. Environmental Factors

Several environmental factors, including pollutants, affect fertility through epi-/genetic routes [16]. The epigenome links the genome and environment and can propagate epigenetic tags across generations [17]. Several recent studies have addressed the sperm-specific epigenetic signature, its transfer to oocytes, and effects on embryo development [17]. For instance, occupational exposure to harmful physical and chemical agents is associated with an increased risk of male infertility, poor semen quality, and decreased motile sperm count [18,19]. Prolonged sitting, exposure to high temperatures (e.g., bakeries and metallurgical industries), or high stress levels can also affect fertility. Job demand or workload is positively correlated with early andropause besides psychological, somatic, and sexual symptoms [20].

Other environmental factors include radiation exposure through mobile phones/laptops, tight-fitting underwear, recurrent hot baths/saunas, and exposure to endocrine-disrupting chemicals (i.e., pesticide residue, bisphenol A, phthalates, and dioxins) [21]. Recent meta-analyses reported a relationship between mobile phone exposure (especially when positioned close to the genitalia) and reduced sperm motility and viability [22,23].

The human microbiome participates in both health and disease regulation through endocrine, circadian, and molecular interactions. Microbial dysbiosis is a risk factor for most non-communicable diseases. Some associations between the microbiome and male reproduction have been reported, though the mechanisms remain ambiguous [24,25]. Either testicular and/or gut microbiome-induced immune system activation may lead to epididymal inflammation and perturbed hormone secretion, including that of leptin, ghrelin, LH, FSH, and testosterone, affecting both spermatogenesis and erectile function [26].

### 2.3. Lifestyle

Lifestyle encompasses all behavioral factors affecting health, including diet, exercise, and the consumption of harmful substances (e.g., tobacco and alcohol). Diet-induced obesity, for example, can affect male fertility by altering sleep and sexual behavior, hormonal profiles, scrotal temperatures, and semen parameters; the risk of a non-viable pregnancy is high for obese men [27]. Moreover, the risk of azoospermia sperm is high in both underweight and overweight men compared to normal-weight counterparts. Decreased sex-hormone-binding globulin levels have been reported in obese men, resulting in hyperinsulinemia and elevated total estradiol levels; contrastingly, weight-loss programs have been associated with reduced cellular DNA damage, increased total motile sperm count, and improved semen morphology [28].

Nutritional habits, alcohol and tobacco consumption, recreational drug usage, and psychological stress affect fertility (Table 1) [21]. Through gut microbiota composition alteration, a high-fat diet can induce intestinal dysbiosis and impede fertility through elevated blood endotoxin levels, inflammation, epididymitis, and dysregulated gene expression in the testes [29]. High-energy and nutritionally poor processed foods have been associated with asthenozoospermia risk, whereas a balanced diet (e.g., Mediterranean diet) is associated with better sperm quality. Lifestyle modifications, particularly on the quality of food consumed, are recommended besides common prescriptions to treat poor semen quality [30].

High alcohol intake and smoking have adverse effects on several sperm parameters [41], reducing fecundity and transmitting epigenetic aberrations to the offspring [16]. Excessive consumption of caffeine and recreational drugs, such as cannabis, opioids, and anabolic steroids affect male fertility [41]. Other crucial lifestyle factors impeding reproductive function in men include lack of physical activity, exposure to stressful conditions, and lack of sleep [42].

### 2.4. Aging and Male Infertility

Aging, a complex multifactorial process, progressively impairs cellular function and promotes vulnerability to diseases. It is associated with disturbances in reproductive endocrinology that potentially causes andropause or late-onset hypogonadism in males [20,43]. However, the molecular underlying mechanisms impacting semen quality and common test parameters are poorly understood. Although the global mean paternal age is 21 years, the most widely referenced cutoff age for advanced paternal aging or andropause is 40 years [6]. Andropause increases infertility risk and affects semen volume and both sperm morphology and motility. However, the effects of aging on sperm concentration remain unclear [44].

Andropause increases the risk of spontaneous abortions and complications in infancy, including lower birth weights, genetic diseases, schizophrenia, and autism [45,46]. Aging can induce several cumulative molecular and/or cellular events, including DNA damage and sperm telomere shortening, leading to cellular senescence or apoptosis [43,47]. Andropause is associated with the accumulation of de-novo mutations, male infertility, and increased genetic risk in the offspring. Telomerase dysfunction seemingly induces a DNA damage response during senescence. However, the effects of andropause on sperm DNA damage remain controversial [48]. Andropause also suppresses the antioxidant defense system and DNA repair machinery, increasing reactive oxygen species (ROS) production and possibly causing genomic instability [49], which can lead to gene expression dysregulation and microRNA (miRNA) patterns [48], both of which are key regulators of normal spermatogenesis.

The multiple facets affecting male infertility (Figure 1), deeply embedded in genome–lifestyle–environment crosstalk, complicate accurate diagnostics development and effective therapeutics. The increasing rates of male infertility highlight the need for integrative approaches that address its complex etiology.

## 3. Markers of Male Infertility

Given the complex etiology of male infertility, several factors possibly interact. In the era of precision medicine, more comprehensive biomarker sets, combining conventional parameters (e.g., sperm morphology, seminal fluid parameters, and DNA damage) and omics-technology-driven markers (e.g., mutations, single-nucleotide polymorphisms (SNPs), transcripts, proteins, and metabolites), are required to elucidate the molecular and pathophysiological basis of male infertility. Hence, improved molecular stratification using effective testing approaches can be implemented towards developing more accurate diagnostics and effective therapeutics.

### 3.1. Seminal Fluid Parameters and Sperm Morphology

**Figure 2:**
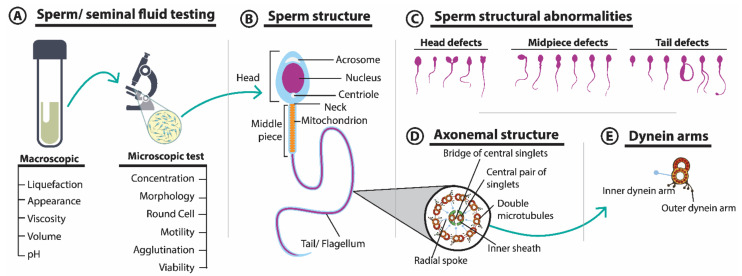
Standard analysis of sperm parameters. (**A**) First-line markers of male infertility diagnosis in seminal fluid and sperm morphology. (**B**) Structure of normal sperm. (**C**) Abnormal morphology due to defects of the head, midpiece, or tail of the sperm. (**D**) Conserved axonemal structure of motile cilia and flagella with a ring of nine microtubular doublets and a central pair of microtubules. (**E**) Inner and outer dynein arms.

Sperm morphology and seminal fluid parameters are considered primary morphological and physicochemical diagnostic markers of male infertility and are crucial for the development of suitable treatments. To avoid inter-laboratory bias, the WHO published a standardized laboratory manual in 1980 for the examination and processing of human semen; the most recent revision was published in 2021 and includes the latest techniques (https://www.who.int/news/item/27-07-2021-who-launches-updated-manual-to-ensure-high-quality-testing-of-human-semen-in-clinical-and-research-settings, accessed on 21 February 2022). According to these guidelines, the two primary quantifiable attributes are spermatozoa number and the fluid volume secreted by various accessory glands [5], though several other microscopic determinants exist (Figure 2A). According to the guidelines, the main semen parameters used in the diagnosis of infertility are: (i) liquefaction (coagulated semen should liquify in 15–20 min at room temperature), (ii) viscosity (high viscosity could indicate prostatic dysfunction), (iii) volume (after 3 to 5 d of sexual abstinence, the average ejaculate volume should be 1.5 to 6 mL, while higher and lower volumes indicate hyperspermia and hypospermia, respectively), (iv) color (normal semen is pearl white and slightly yellowish), (v) pH (must be >7.1, lower values could indicate efferent vessel dysgenesis that leads to low sperm concentration), (vi) concentration (15 million spermatozoa per milliliter of ejaculated volume), (vii) motility (the proportion of motile spermatozoa should be >32%), (viii) vitality (proportion of live spermatozoa must be >58%), (ix) leukocyte concentration (more than 1 million/mL of sample), (x) morphology (≥4% of spermatozoa in a sample should be normal), and (xi) anti-sperm antibodies (according to a mixed antiglobulin reaction (MAR) test, attachment of ≥50% of spermatozoa to other cells or particles indicates an immune disorder) [5].

Advanced microscopic tools have enabled in-depth structural investigation of sperm morphology (Figure 2B) and identification of potential abnormalities (Figure 2C). The sperm tail is essential for motility and fertility. Abnormal tail structures may result from tissue-specific gene and protein expression/aberration [50,51]. Motile cilia malfunction causes primary ciliary dyskinesia, a genetic condition (briefly described in Section 3.4) associated with sperm phenotypic defects. While ciliary structural defects can be identified by transmission electron microscopy, both ciliary beat patterns and frequency defects can be identified by high-speed video microscopy analysis [52].

### 3.2. Reactive Oxygen Species

Approximately 30–80% of men with idiopathic infertility show increased concentrations of free oxygen radicals or ROS [53], a candidate marker for male infertility. OS occurs when ROS levels increase disproportionately to antioxidant-neutralizing capacity. In the male reproductive system, ROS can be derived from sperm cells, though leukocytes produce at least 1000-times more ROS than spermatozoa. Approximately 10–20% of infertile men have an increased number of leukocytes in the ejaculate [5], but this is likely underestimated, given the relatively high cutoff value for leukocytospermia (>106/mL). The accuracy of the clinical cutoff value for leukocytospermia remains controversial due to conflicting data on the physiological and pathological roles of leukocytes in semen samples [54].

Although free radicals control sperm maturation, capacitation and hyperactivation, acrosome reaction, and sperm–oocyte fusion, they can also initiate protein damage, lipid peroxidation, DNA damage, and apoptosis [55]. Both high and low levels of OS can affect sperm function by impairing viability, motility, and fertilization potential [14,15,56], with sperm being particularly susceptible to OS due to high polyunsaturated fatty acid (PUFA) concentrations in their plasma membranes, a lack of antioxidant defense, and limited cell repair systems.

### 3.3. Sperm DNA Fragmentation (SDF)

DNA damage/fragmentation represents an alteration in the DNA structure that causes cellular injury and reduces cell viability. As a major molecular cause of male infertility, sperm DNA fragmentation (SDF) has become an important prognostic and diagnostic marker [57] and correlates well with conventional semen parameters, including abnormal head shape and reduced progressive motility [58]. The assessment of SDF also offers a tool for selecting sperm with the best DNA integrity for use in ARTs [58].

**Figure 3:**
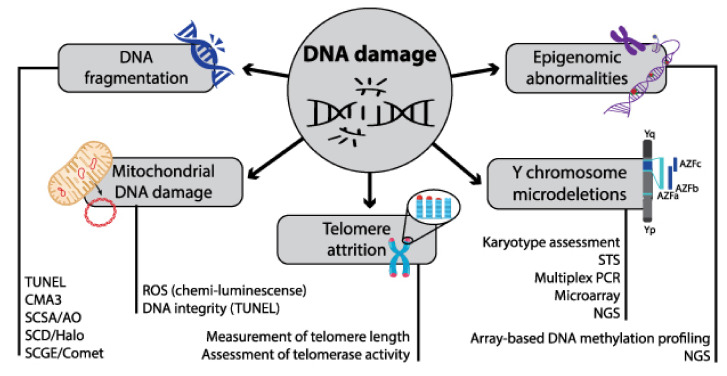
Different types of DNA damage and their possible methods of assessment.

DNA damage can broadly be classified into two categories: endogenous and exogenous. Endogenous DNA damage arises from naturally present factors or chemicals; exogenous DNA damage is induced by foreign agents or factors [59]. The main endogenous DNA damage types caused by ROS include (i) DNA fragmentation, (ii) mitochondrial DNA damage, (iii) telomere attrition, (iv) Y chromosome microdeletions (Y-CMs), and (v) DNA methylation and acetylation (epigenetic factor) (Figure 3). DNA fragmentation can occur on either single- or double-stranded DNA. ROS induces DNA fragmentation by modifying DNA bases and inducing the release of 8-hydroxy-2′-deoxyguanosine, a marker of DNA fragmentation [60]. Unlike genomic DNA, circular mitochondrial DNA are more vulnerable to ROS given their lack of histones and protamines. Since mitochondria produce ATPs, mitochondrial dysfunction leads to higher ROS production [61]. Telomeres contain non-coding DNA repeats and protect chromosomal DNA from degradation by ROS. The shortening of telomere repeats indicates cellular aging. Epigenetic modifications in methylation and acetylation processes induced by ROS result in harmful effects on sperm production and function [62].

DNA replication errors and base mismatches can occur during cell division. These errors or mismatches escape proofreading and mismatch repair (MMR) pathways and become mutations in the ensuing replication round or result in DNA damage [63,64]. Topoisomerase enzymes, which remove super-helical tension from DNA during replication and transcription, can cause endogenous DNA damage. Base deamination is another source of mutation that converts cytosine, adenine, guanine, and 5-methyl cytosine to uracil, hypoxanthine, xanthine, and thymine, respectively, by removing the exocyclic amine group. Unstable basic sites are continuously generated in DNA when the N-glycosyl bond is cleaved or hydrolyzed, which may influence endogenous DNA damage [59]. Besides endogenous DNA damage and epigenetic changes, Y-CMs are an important cause of male infertility [65].

Several assays are used to assess SDF, despite varying results between tests (Table 2). Based on their ability to measure sperm chromatin integrity or DNA damage, they are classified into direct and indirect tests. The terminal deoxynucleotidyl transferase nick-end labelling (TUNEL) assay is recommended, given its ease of use, and allows for stronger correlations with embryo viability. Moreover, the TUNEL assay is robust and highly reliable in identifying both single- and double-strand DNA breaks in spermatozoa from neat, washed, and cryopreserved semen samples [66].

Several techniques are used to assess sperm DNA fragmentation, including: (i) aniline blue staining and the chromomycin A3 test, (ii) sperm chromatin structure assay (SCSA), (iii) sperm chromatin dispersion (SCD) test, (iv) comet assay or single-cell gel electrophoresis (SCGE), and (v) DNA-breakage detection-fluorescence in-situ hybridization [67,68] (Table 2).

However, these tests do not reveal the type and location of DNA damage. Therefore, high-throughput DNA sequencing platforms are recommended for improved specificity, accuracy, coverage, and discovery of DNA fractures, microdeletions, and SNPs, besides improved statistical power in infertility status analysis.

### 3.4. Genomic Markers

Approximately 2000 protein-coding genes contribute to the genesis and maturation of millions of male gametes, which takes 72 days to complete. Therefore, the genetic landscape of male infertility is highly complex and is an emerging area of research. Genetic factors impact all major etiological categories of male infertility, and some can be tested by routine diagnostics [69]. Genetic factors have been identified in 10–20% of spermatogenic impairment cases, though the majority of these gene–disease relationships require verification [70].

**Figure 4:**
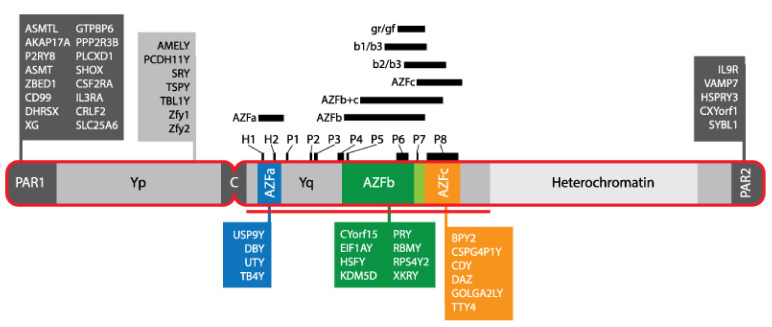
Structure and genes of the Y chromosome. Genes of each region are listed in a color-coded box with corresponding segments. The pseudoautosomal region and centromere (C) are shown in dark grey. The short arm (Yp) and long arm of the Y chromosome (Yq) are shown in light grey. AZF (-a: blue, -b: green, -c: orange, -b/c overlapping region: lime green), azoospermia factor; H1, HERV15yq1; H2, HERV15yq2. The palindromic regions (P1 to P8) are shown above the chromosome in black alongside various Y chromosome deletions.

Although karyotype is the oldest known genetic testing of azoospermia/oligozoospermia, Y-CMs have become increasingly relevant genetic causes of male infertility, thanks to the development of potent molecular analysis tools. Y-CM is more prevalent in spermatogenic failure than in normochromic men, and occurs in 5% of oligozoospermic men and 10% of men with azoospermia [71]. The vast majority of these are de-novo microdeletions, i.e., microdeletions that occur as cellular events during spermatogenesis, indicating that the Y chromosome is particularly unstable. The Y chromosome is acrocentric, with a short arm (Yp) and a long arm (Yq) (Figure 4). During meiosis, only pseudoautosomal regions of the Y chromosome undergo recombination with the X chromosome, whereas the male-specific region, which comprises 95% of the Y chromosome and contains 78 protein-coding genes, does not. Among them, 27 genes are involved in spermatogenesis and testis development, among other organs [72] (Figure 4). Frequent microdeletions in the azoospermia factor (AZF) region of Yq are associated with spermatogenesis failure. There are three distinct regions in AZF, AZFa, AZFb, and AZFc, each containing various genes for a variety of functions. AZFa is located most proximally from the centromere, followed by AZFb and, most distally, AZFc [73]. Severe deletions of AZFa and AZFb are not transmissible, while men with AZFc deletions will commonly require ART. Therefore, individuals with azoospermia and severe oligozoospermia are recommended to undergo Y-CM screening and karyotype assessment, according to the American Society for Reproductive Medicine guidelines [74]. Currently, the molecular diagnosis of Y-CM involves PCR-based analysis of sequence-tagged site markers that are mapped within specific AZF regions of the Y chromosome. Contrastingly, routine PCR may fail to identify novel Y-CMs or microduplications. Hence, a higher-resolution analysis of all the Y chromosome loci is required in order to simultaneously assess its integrity in a single assay. A new microarray procedure targeting known Y-CMs that are undetectable using conventional multiplex PCR technologies has recently been developed [75]. However, multiplex PCR is the most commonly applied Y-CM detection method and is used to amplify small portions of each region, with losses reported only as AZFa, AZFb, and/or AZFc deletions [65,76]. Recently developed panels for male/female infertility genes achieved high accuracy in diagnosing copy number variants (CNVs), insertion/deletions, sex chromosome aneuploidies (94% accuracy for Y-CM), cystic fibrosis transmembrane conductance regulator (CFTR) gene, and thymidine tract length quantification [65,76].

The X chromosome does not undergo replication during meiosis; therefore, it is seemingly protected from unpaired chromosome inactivation, similar to the Y chromosome. Although the X chromosome may have an important function in germ cell survival, X-linked palindromic genes might not be essential for spermatogenesis [77]. Most single-copy genes of X chromosomes are conserved among species, which complicates the study of these genes in animal models. Furthermore, validated X chromosome-linked monogenic causes of male infertility are surprisingly uncommon [78], with a few exceptions: (i) aneuploidy of the X chromosome in Klinefelter syndrome, (ii) X-chromosome or X-autosome translocations (XX-male syndrome), and (iii) point mutations disrupting X-chromosomal genes [79]. Klinefelter syndrome is a chromosomal condition caused by the presence of an extra X-chromosome. Approximately 0.2% of the general population has this syndrome, compared to 15–20% of nonobstructive azoospermia (NOA) patients [78,79].

CNVs are structural variants with changes in the number of copies of specific DNA regions compared with the reference genome. CNVs are major causes of human genome variability due to deletion or duplication of the original sequence, without any additional mutation, resulting in unequal crossover between or within chromosomes. Quantitative spermatogenic disturbance analysis revealed that X-linked CNVs were associated more with infertile men than controls [80]. For example, CNV67 deletion affects *MAGEA9*, a gene on the X chromosome that is specifically expressed in the testis under physiological conditions [78]. Similar to the X chromosome, there is a strong belief that several other autosomal loci may impair male fertility as a consequence of CNVs [81]. Indeed, some gene mapping on the Y chromosome seems to directly affect male fertility [73]; thus, any chromosomal anomaly causing under-expression or loss of function can impair male fertility. From this perspective, the SRY and AZF loci can be considered models. However, more complex contexts with additional copies of one or more genes can be expected, such as aneuploidies, duplications, or unbalanced translocations. There is little evidence supporting a direct relationship between CNVs and other male-sterility-related genes, such as *RBMY1* and *DAZ*. However, other male-fertility-related Y-linked genes may be involved, given that some show clear up- or down-regulation in infertile men [80].

Gene polymorphisms are genomic variations, in which two or more discontinuous genotypes or alleles are simultaneously present in a population. SNPs are variations caused by mutations at a single position in a DNA sequence. Disease-associated genetic variants are highly penetrant monogenic variants—a single gene mutation leading to a consistent disease phenotype. Although these variants may be associated with the disease, they do not directly affect gene function [82]. The majority of male-infertility-associated genetic variants are located on sex chromosomes [82,83,84]. Other autosomal polymorphisms have also been identified. For example, SNPs in methylene-tetra-hydro-folate reductase (MTHFR), a key enzyme in folate metabolism, contribute to an increased risk of male infertility [85,86]. Another gene that encodes DNA polymerase gamma (POLG), an enzyme responsible for the replication and repair of mitochondrial DNA, is also associated with sperm dysfunction; however, the role of POLG in male infertility remains controversial [87,88].

The MMR pathway plays a critical role in the maintenance of genome integrity, meiotic recombination, and gametogenesis. SNPs in MMR genes reduce MMR function and may lead to mutations in other genes. SNPs (MLH1, MSH2, PMS2, MLH3, MSH4, MSH5, and MSH6) in MMR genes result in male infertility [63,64,89]. The MMR gene *MLH1* is involved in spermatogenesis and is associated with male infertility (i.e., oligozoospermia), likely through epigenetic regulation (i.e., methylation) [90].

Mitochondrial genes are the key molecular components of sperm cells. Mature mammalian spermatozoa contain large amounts of mitochondria required for energy production to support motility. Mitochondria also regulate several pathways involved in spermatogenesis [91]. A higher prevalence of 4977 mtDNA was found in subjects with impaired sperm motility and fertility, indicating that the maintenance of the mitochondrial redox microenvironment and genome integrity influence sperm function regulation [92]. Although 785 point mutations have been identified in the non-coding control regions, rRNA genes, tRNA genes, and the coding regions of mtDNA samples, which were mainly transition mutations, identifying the roles of these genes in male fertility requires further investigation [93].

### 3.5. Transcriptomic and Epigenomic Markers

Spermatozoa are considered sophisticated paternal-genome-delivery vehicles that contain several nucleic acids (DNA and RNA) in their cytoplasm [94]. More than 270 types of RNAs have been reported in mature human spermatozoa and their functions in embryo development remain unclear [95]. Interestingly, seminal plasma RNAs influence the sperm RNA content, which is modulated during epididymal transit. Spermatozoa in the caput epididymis are enriched with miRNAs, while tRNA-derived fragments are more abundant in the cauda. Spermatozoa retrieved from the caput epididymis were unable to penetrate the oocyte, possibly due to a lack of competence/capacitation for fertilization provided by the RNAs, proteins, and metabolites of the cauda [96]. Differential expression of miRNAs has been observed in the seminal plasma of fertile and infertile men [97,98]. Additionally, efforts are also diverted towards the identification of differentially expressed circular RNAs as possible epigenetic regulators/markers of spermatic function and sperm quality [99]. Although further validation is needed, some potential miRNA markers that may facilitate accurate male infertility diagnosis and treatment have been reported (Table 3). Collectively, these findings support the importance of the seminal plasma transcriptome in fertility.

Epigenomics is the main route of environmental impact on male (in)fertility [17]. DNA methylation is an epigenetic factor that plays a critical role in spermatogenesis [100,101]. Proper methylation ensures successful chromatin condensation in the sperm head, enabling sperm maturation and regulating fertilization and post-fertilization events [102,103]. Several studies have analyzed the association between male infertility and methylation of sperm DNA [104]. For example, the impairment of MTHFR by methylation can contribute to diseases, including male infertility [105].

**Table 3:** Transcriptomic and epigenetic factors associated with male infertility.

miRNA/Transcriptomic and Epigenomic Factors	Regulation	Association with	Ref
miR-196a-2, miR-196a-5p, miR-141, miR-429, and miR-7-1-3p	Up-regulation	Idiopathic male infertility	[97,106]
miR-424	Down-regulation	Idiopathic male infertility	[107]
MiR-371a-3p	Up-regulation	Sperm concentration and total sperm count	[108]
piR-31068, piR-31098, piR-31925, piR-43771, and piR-43773	Differentially expressed/ down-regulation	Asthenozoospermia	[109]
miR-19b and let-7a	Up-regulation	Idiopathic infertility	[110]
hsa-let-7b-5p	Down-regulation	Asthenozoospermia/idiopathic male infertility	[111]
miR-192a	Up-regulation	Germ cell apoptosis	[112]
miR-23b, miR-146a, miR-155, miR-223, miR-17-92, and miR-34a	Down-regulation	Miscarriage, pre-eclampsia, and small for gestational age fetuses	[113]
MTHFR promoter	Hypermethylation	Abnormal concentration/motility of sperm	[114,115,116]

Histones are suitable candidates for the transmission of epigenetic information, given their involvement in chromatin folding and transcription regulation [117]. Aberrant H4 acetylation is associated with impaired spermatogenesis and Sertoli cell-only syndrome in infertile men. Other epigenetic alterations that involve changes in factors that regulate gene expression have also been associated with various conditions and disorders, including abnormal sperm profiles in infertile men [62].

The emergence of high-throughput techniques has enabled exploration of the relationship between DNA methylation and male infertility [118]. These genome-wide association (GWAS) studies could help investigating the changes in methylation patterns in the male reproductive system, either in fertile or infertile men, to identify spermatogenesis-related genes and reliable biomarkers [62]. An array-based DNA methylation profile using peripheral blood from infertile men can also be considered for diagnostic purposes [119]. However, these approaches require large and multicentric studies to identify benchmark biomarkers with tangible outcomes.

### 3.6. Proteomic and Metabolomic Markers

The spermatozoon is a highly specialized and easily accessible cell. Therefore, it is remarkably suitable for proteomic analysis, as a whole cell or isolated organelles, of the expression of functional and structural proteins, during either spermatogenesis or spermiogenesis and all their post-translational modifications. Other techniques, including Western blotting and ELISA, are used to identify targeted proteins. Despite the progress made in understanding some molecular events associated with sperm maturation and fecundity, additional studies are required to unravel the pathophysiology of male infertility at the proteomic level [120,121].

Proteomics of mature sperm cells generally reveals two types of proteins: (i) proteins of extracellular origin (i.e., accessory sex glands), adsorbed on the surface of the ejaculated sperm cell, such as seminogelin-1 and prostate-specific antigen (PSA), and (ii) sperm cell proteins divided into detergent soluble and insoluble fractions. The detergent-soluble fraction comprises proteins in the cytoplasm, signaling molecules, and membrane receptors, whereas the detergent-insoluble fraction comprises cytoskeletal/structural and nuclear-chromatin-binding proteins. Up to 11% of sperm proteins participate in cell defense against OS and apoptosis. Therefore, the differential expression of these protective factors in the sperm of infertile men with leukocytospermia may explain the generation of OS in these patients. Additionally, several proteins that correlate with sperm DNA integrity have been identified and can serve as markers to discriminate obstructive from nonobstructive azoospermia [122,123]. Clusterin, epididymal secretory protein E1, and PSA have been proposed as seminal biomarkers for in-vitro fertilization (IVF) success in unexplained infertile couples [124] and correlated with sperm quality, motility, and viability [125].

Metabolomics is another high-throughput technology used to study disease mechanisms and diagnosis, with seminal fluid, serum, and urine samples being commonly used for metabolomic fingerprint research in male infertility [126,127]. A seminal plasma metabolic signature study demonstrated that environmental exposure to arsenic, phthalate esters, and perfluorinated compounds was associated with poor semen quality [128]. Metabolomics can also function as an infertility diagnostic tool [129,130], and about 44 metabolites were differentially expressed in infertile men [131]. Interestingly, these metabolites predicted infertility with a specificity of 92%.

Despite efforts to use omics technologies in identifying clinically actionable biomarkers, the studies are scattered and performed mostly at an institutional level, therefore, requiring multicentric validation and association with other omics and clinical settings to be translated safely and effectively. High-throughput technologies are required to study the genomic, transcriptomic, proteomic, metabolomic, and metagenomic profiles of infertile men and their association with sperm DNA damage, inflammation, and ROS using appropriate controls (fertile donors).

## 4. Current Therapeutic Options

So far, no pharmacological treatments for stimulating spermatogenesis in primary testicular failure have been approved. The main therapeutic option for infertile men is assisted reproductive technologies (ARTs) followed by surgery.

### 4.1. Assisted Reproductive Technologies (ARTs)

In the USA, ~1% of successful births were attributed to ARTs in 2001. ARTs encompass ovarian stimulation, sperm retrieval, in-vitro gamete assessment, intrauterine insemination (IUI), intracytoplasmic sperm injection (ICSI), gamete and/or embryo cryopreservation, and IVF. Other procedures, such as preimplantation genetic diagnosis and -screening (PGD and PGS), are also considered adjunctive tools for ART. In the absence of evidence-based science, management of male factor infertility relies extensively on ARTs. IUI is the primary option when the female partner is fertile and enough motile spermatozoa (>10^6^ motile cells) can be retrieved. When >3 cycles of IUI fail, optimized in-vitro fertilization (IVF) using ICSI is usually recommended. Sperm cells, in this case, are recovered either surgically or from seminal fluid [132].

Although ART is the main procedure for effective subfertility treatment, its availability, accessibility, and affordability differ between countries [133,134], and post-treatment complications remain a concern [135]. Some common complications include ovarian hyperstimulation syndrome, a risk of multiple pregnancies, and low birthweight [136,137,138]. Therefore, the development of safe and cost-effective therapeutic options to address male infertility is necessary in this post-genomic era.

### 4.2. Surgical Approaches

Surgical approaches, including robotic surgery, are increasingly used to treat particular types of male infertility. Men with obstructive azoospermia can be treated with epididymal or testicular sperm extraction (TESE), using microsurgical epididymal sperm aspiration (MESA), percutaneous epididymal sperm aspiration (PESA), or reconstructive surgery. TESE/micro-TESE can retrieve testicular sperm in up to 50% of NOA patients [139]. However, mean serum testosterone levels were reduced after six months of TESE [140]; thus, endocrine surveillance for hypogonadism should be considered in men with NOA after TESE. Furthermore, genetic disorders, such as AZFa/AZFb microdeletions or XX male syndrome, are contraindications to TESE because of their incompatibility with spermatogenesis.

Reconstruction of the testes and scrotum for male infertility treatment may fall under simple hydrocelectomy; however, this could be complex in some cases. In adults, hydroceles were found in 1% of fertile men, while 0.7% were found in infertile conditions. In the primary stage, tetracycline or phenol is injected into the hydrocele, a technique known as sclerosing or sclerotherapy, which can alleviate 60–90% of scrotal problems. This constitutes a simple option for removing hydroceles and improving male fertility [141].

Although conventional penile reconstruction has many disadvantages [142], advanced microsurgery facilitates phalloplasty technique, including a radial artery forearm flap, thigh flap, and latissimus dorsi flap. The radial forearm is the most commonly used technique, with 80% of patients reporting improved sensation, 99.1% reporting normal urination, and 98% reporting satisfactory outcomes [143]. Most importantly, 75% of the treated population can achieve orgasm [144], though some patients require post-surgery anastomotic revision. A fibular osteocutaneous flap can provide long-term rigidity to the penis that allows for normal sexual intercourse [145]. Disadvantages of this technique include partial flap loss (~12% of total cases) and fistula formation [146]. Another advanced approach in penile reconstruction is an anterolateral thigh flap, where 100% of patients report improved sensation.

### 4.3. Antioxidants

Antioxidants can scavenge free radicals and treat OS in infertile patients [147]. The therapeutic use of enzymatic antioxidants, such as superoxide dismutase (SOD), is limited due to its high instability, low half-life, and high immunogenicity [148,149]. Catalase (CAT), another antioxidant, assists in the conversion of H_2_O_2_ into molecular oxygen and water; however, its usage and effect in human sperm remain uninvestigated [149]. Glutathione peroxidase (GPX) is a CAT that may influence human fertility, with higher sperm recovery, motility, and bioavailability after cryopreservation [150]. Another promising enzymatic antioxidant is inositol, which shows improved sperm parameters [151,152].

A non-enzymatic antioxidant group, obtained either through endogenous metabolism or diet, can be used to address male infertility (Table 4). This group includes Q-10 coenzyme (CoQ10), carnitine, and lycopene. CoQ10 reduces ubiquinol by oxidizing ubiquinone and protects the cell membrane from lipid peroxidation; CoQ10 oral supplements significantly improved sperm concentration and motility [153]. Carnitines are long-chain fatty acid transporters in the mitochondria that contribute anti-apoptotic effects, which have a positive relationship with sperm quality [149]. Lycopene, a primary carotenoid found in the testes at high concentrations, has antiproliferative, immunomodulatory, and anti-inflammatory effects, which promote cell differentiation, improve sperm count, decrease seminal OS, and increase IVF success rates [154].

Other antioxidants, such as N-acetylcysteine (NAC), melatonin, alpha-lipoic acid (ALA), and omega-3 fatty acids (OFA), can also be applied in fertility management. NAC, a precursor of GPX, can directly stabilize free radicals by donating an electron from its outer layer. Multiple studies involving NAC have shown that it improves male fertility by increasing seminal fluid [163], reducing ROS molecules in sperm [177], and improving other sperm parameters [149]. Melatonin is an amphiphilic hormone that increases SOD, CAT, and GPX activities [165] to scavenge ROS [178], and even abolish apoptosis [165]. Fertile men show higher seminal and serum levels of melatonin than infertile men [167], and melatonin levels correlate with DNA fragmentation and sperm viability [165,167]. ALA is another potent biological antioxidant that can enter the Krebs cycle and assist in ATP production, promoting the functionality of SOD, CAT, and GPX [170]. Oral supplementation with ALA or cell incubation improved sperm quality parameters, such as total sperm count, concentration, motility, viability, and sperm morphology [170,172,173,179]. Finally, OFA intake increased normal sperm morphology, volume, concentration, motility, and total sperm count [175,180].

### 4.4. Vitamin and Mineral Supplementation

Vitamins play an essential role in the normal functioning of the human body, with vitamins C, E, and B9 (folic acid) being the most relevant in male fertility (Table 5). In sperm cells, vitamin C prevents agglutination, protects against DNA damage caused by ROS [181], and improves sperm parameters [182]. Vitamin E serves multiple functions in male fertility, including regulation of testosterone biosynthesis, telomerase activity [183], and lipid peroxidation activity. Folic acid is essential for DNA metabolism and gene expression to prevent abnormal chromosomal replication and mitochondrial DNA deletions; however, its role as a suppressor in improving male infertility requires further exploration [184].

Minerals, especially zinc and selenium, influence male fertility. Zinc is a micronutrient that participates in cell signaling, enzyme activity, normal growth and sexual maturation regulation, and management of mitochondrial OS. Zinc incorporation into sperm may protect against sperm decondensation and alleviate sperm motility, membrane stabilization, antioxidant capacity [195,196], and normal sperm morphology. Low zinc levels are widely reported in the seminal plasma of infertile men [197,198]. Selenium targets free radicals, alleviates testicular toxicity, promotes DNA repair [199], and is positively associated with sperm count, morphology, motility, and concentration [190,192,200]. Higher levels of successful conception and live births were correlated with higher seminal selenium levels [194].

### 4.5. Hormonal-Based Therapies

Hormone-based therapy involves the use of hormones or their antagonists in medical treatment. Hormone therapy improved endogenous follicle-stimulating hormone and/or androgen levels and, subsequently, spermatogenesis in infertile men [201]. Gonadotropin replacement therapy and antiestrogens are administered to azoospermic men before surgical sperm retrieval, although their efficacy is lacking. Gonadotropin therapy is highly effective but not necessarily in men with idiopathic oligozoospermia. Improved birth and pregnancy rates were observed in males receiving follicle-stimulating hormone [202]. However, a lack of standardization exists in the treatment duration and dose/type of antiestrogen therapy. Moreover, the use of these pharmacological therapies for testicular failure pre-ICSI or -TESE is still controversial and not supported by current guidelines [203]. Furthermore, antiestrogen therapies may have some side effects on male sexual function (sexual desire and erectile function) [204].

## 5. Promotion of a Healthy Lifestyle: A Promising but Underexplored Approach

Selective lifestyle choices are cost effective, accessible, and useful as male infertility prevention and treatment tools. The duration of infertility, age of the couple, and comorbidities are among the main factors influencing spontaneous conception [205]. Frequent sexual intercourse (>3 times per week) can increase the likelihood of conception. Approximately 30% of couples in whom the male partner has a sperm concentration of 1 to 5 million/mL will conceive spontaneously over 24 to 36 months. A low sperm concentration of 1 million/mL does not preclude natural fertility, though the chances decrease over time as sperm defects, co-existing exposure, and disease increase with age [206].

Lifestyle changes associated with a healthy diet represent a potentially important treatment for male infertility (Table 6). A high body mass index (BMI) is negatively correlated with male fertility and bariatric surgery is an effective weight-loss therapy; however, despite normalizing the reproductive hormone profile, it may not affect sperm function within two years post-surgery [207]. Similarly, milder weight loss is associated with improved sperm function in obese men, increased sperm count, motility, semen volume, and testosterone, and reduced SDF [28,208]. BMI can generally be improved by consuming a healthy diet and engaging in regular physical exercise. Resistance training has been shown to improve fertility in men. Adequate sleep and mindful living are crucial for general well-being and also affect reproductive health. However, the crosstalk between reproductive hormones and sleep patterns is bidirectional in its effects on fertility and, thus, more complex than is currently understood. Finally, amelioration of adverse lifestyle factors, such as alcohol consumption and smoking, can also enhance male fertility outcomes [16,30,41].

## 6. The Potential of Multiomics

In the post-genomic era, advanced multiomics and digital approaches have revolutionized biomedical research. These omics technologies have allowed unprecedented resolution of molecular processes, as well as the accurate diagnosis and molecular stratification of diseases, including idiopathic male infertility. Advances in whole-genome and whole-transcriptome amplification have expedited the sequencing of minute amounts of DNA and RNA from a single cell and provide a more representative scope of the nature of genomic and transcriptomic heterogeneity that occurs in both normal and diseased cells [229].

Stem cell therapy has recently emerged as a new approach to infertility management. In addition, advanced cell culture technology and in-vitro cell proliferation models allow somatic cell use in infertility treatment [230]. Advances in single-cell omics techniques are accelerating the elucidation of male infertility mechanisms and malfunctioning affecting spermatogenesis [230]. A recent review discussed the usefulness of various stem cells in male infertility treatment [231]

Male infertility microsurgery has significantly progressed, with new and emerging techniques, technologies, and methodologies being continuously developed [232]. Robotic surgery offers improved visualization, ergonomics, and tremor reduction [233]. The use of artificial intelligence, deep learning, and machine learning have been widely applied in urologic oncology and show great potential in the study of infertility treatment [234,235].

Among others, genomics, transcriptomics, epigenomics, proteomics, metabolomics, reactomics, pharmacogenomics, and bioinformatics are particularly relevant “spermomics”/multiomics technologies in the assessment of sperm cells and seminal fluids and can enhance our understanding of the molecular events driving spermatogenesis and spermiogenesis in fertile versus infertile men. These approaches provide unprecedented power of data analysis, visualization, interpretation, and compilation [236].

**Figure 5:**
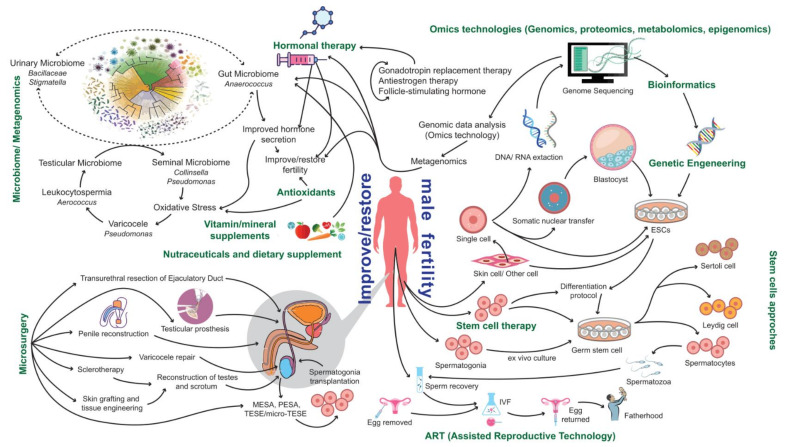
An overview of current advanced approaches to assess and restore male fertility.

In male reproductive medicine, efforts in “spermomics” technologies and associated efforts are scattered, hampering tangible, reproducible, and clinically actionable outcomes (Figure 5). Therefore, the integration of spermomic approaches and microsurgery or robotic surgery could constitute effective theranostic options and allow increased success rates, for induction of spermatogenesis, reconstruction of the reproductive tract, or the retrieval of spermatozoa for assisted conception.

## 7. Advances towards Precision Male Reproductive Medicine

While advanced molecular biology and DNA damage assays allow clinicians to assess idiopathic male infertility, microsurgery has increased the success rates of spermatozoa retrieval for assisted conception. In reproductive endocrinology, PGD and PGS are used for genetic and aneuploidy testing to identify the relevant cause(s) of infertility, provide personalized management, and improve IVF outcomes. Although some individuals with antioxidant/ROS imbalance could be treated with oral supplementation of antioxidants [237], deeper investigation into the hidden roots of idiopathic infertility is necessary to address the effects of potential factors and/or pathogens that reduce sperm concentration and motility and affect morphology [238]. The same integrative and investigative strategy is required for treating pathogenic infections in human ejaculate and associated inflammation (ROS) in the male genital tract by using antibiotics and/or anti-inflammatory agents to prevent the deterioration of sperm parameters [239,240,241].

**Figure 6:**
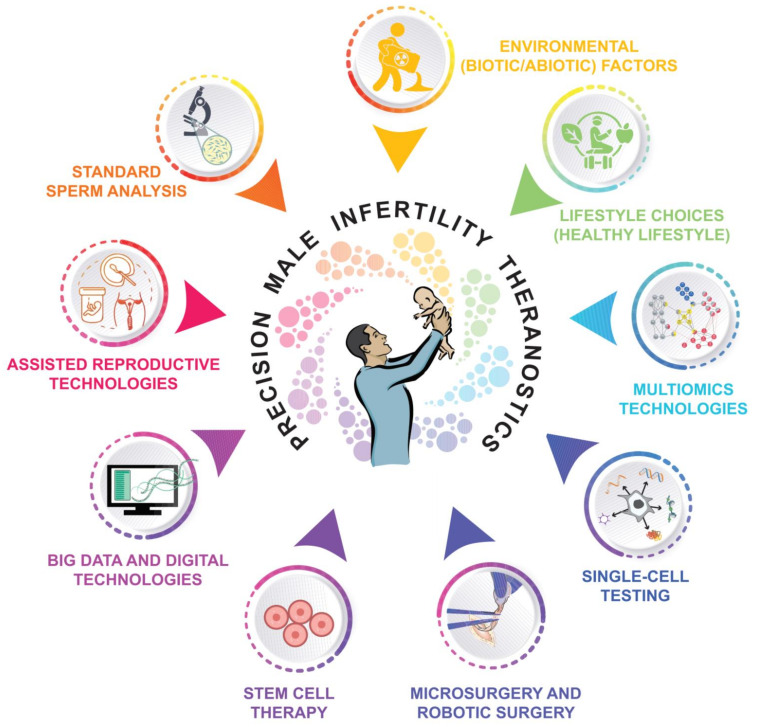
Areas of interest that should be integrated in a comprehensive approach towards precision male infertility theranostics.

Precision medicine aims to provide an effective and individualized treatment plan through a comprehensive data-driven approach based on omics techniques. Personalized molecular treatment is the basis of all ongoing efforts in the development of male reproductive medicine. Demystifying the complex etiology of male infertility requires a comprehensive approach that combines all relevant aspects to achieve precision male infertility theranostics (Figure 6), including standard sperm analysis, robotic surgery, stem cell therapy, ARTs, multiomics analysis technologies, and single-cell testing/imaging. In addition to the add-value of big data and digital visualization technologies, it needs to be emphasized that male infertility theranostics should also integrate individual lifestyle choices and environmental factors as key determinants that complement the clinical efforts. Environmental factors can affect hormonal profiles, testis cell differentiation, sperm maturation, and transport in the epididymis. Nutraceuticals, for example, have been shown to provide additional health benefits, modulate sperm quality parameters, and affect male fertility [196,242]. Dietary habits also determine the composition of gut microbiota, which offers additional direct and indirect preventive and therapeutic options, though the role of human microbiota remains unclear [25,26].

Of note, the progress in male infertility management and treatment have been associated with several ethical issues that need to be addressed, especially that more couples are deferring having children to older ages due to several reasons. In fact, the IVF, ICSI, sperm donation, long-term gametes freezing, posthumous sperm retrieval are ART procedures that have been concomitantly associated with ethical debates, consenting dilemmas, and socio-legal issues. The desire of having a child using the latest technologies, discussed herein and elsewhere, is far from being a smooth decision for couples and/or doctors. The rights and values of couples, their families, and their future children, should be integrated together while considering the values and ideals of the society to reach an informed, concerted, and balanced decision/judgement that maximizes the benefits while minimizing/preventing the harms.

Since the pathophysiology of male infertility is still obscure, it is worthwhile to combine the advanced approaches, especially high-throughput multiomics technologies and big data tools, into comprehensive and large-scale strategies, along with lifestyle choices and environmental factors, in order to develop diagnostic clues, management avenues, and promising therapeutic options towards precision male infertility therapeutics and diagnostics.

## 8. Conclusions

This review highlights the importance of integrative approaches that combine conventional sperm analyses, omics technologies, digital tools, as well as the effects of lifestyle and environmental determinants of male infertility. Sperm cells, along with the seminal fluid, are the host of key biomarkers/bioindicators to assess male fertility power, accurately diagnose possible infertility, and predict potential effects on embryo development and offspring health. Given the increasing burden of male infertility on populations worldwide, global networking and collaboration is urgently needed to establish comprehensive strategies that are representative of the population being treated, consider the most immediate environmental factors, and incorporate the latest advances in analytical technologies, including omics tools, stem cell therapy, robotic surgery, ART, big data, and digital algorithms. Therefore, collaboration across all relevant fields and the involvement of all stakeholders would facilitate the success of future clinical approaches. Incorporation of biological, environmental, socioeconomic, and lifestyle determinants will not only help elucidate the intricate networks that govern susceptibility, causes, and molecular progression in male infertility, but will also advance the characterization of accurate biomarkers, from genes to metabolites, and establishment of comprehensive, proactive, cost-effective, precise, and accessible curative strategies.

Multi-institutional efforts are crucial not only in alleviating the tremendous technical and infrastructural limitations, but also to address cultural, educational, logistic, and socioeconomic limitations. Most importantly, unless the public is educated to improve awareness, break down stigmas, and inform patients of potential curative options and their possible side effects (ethical counselling), no clinical advance will be successful. Additionally, omics-driven medicine training should be provided to a broader range of healthcare professionals to accelerate the application of new diagnostic tools and innovative therapeutic options. Finally, the advent of digital health applications could also equip patients to become proactive in addressing infertility.

## Figures and Tables

**Table 1:** Associations between dietary habits and male fertility.

Nutritional Factors	Findings	References
Dietary patterns	Unhealthy diets (western diet) increased the risk of infertility.	[31,32]
Dietary fats	High-fat dairy products may increase the risk of infertility. Trans fatty acids may increase the risk of metabolic disorders that negatively affect ovarian function.	[33,34,35]
Vegetables and fruits	Although vegetables and fruits were associated with improved semen quality and fertility related to antioxidants, folic acid, fiber, and minerals, pesticide residues may modify the beneficial effects.	[35,36,37,38,39,40]
Beverages	High intake of sugary beverages increased infertility risk.	

**Table 2:** Techniques used to assess sperm DNA fragmentation.

Test	Purpose	Method	Principle	Result
TUNEL	To identify single- and double-strand DNA breaks	Fluorescence-labeled nucleotides are added to the site of DNA fragmentation	Quantifies the enzymatic incorporation of dUTP in DNA breaks	Sperm with fragmented DNA fluorescence
CMA3 staining	To determine DNA integrity	Staining by CMA3	Competes with protamine for the same binding site in DNA	A positive test indicates a low DNA protamination state associated with poorly packaged sperm chromatin
SCSA/AO test	To detect breaks in single-stranded DNA (ssDNA)	Acid denaturation, followed by staining with AO	AO emits fluorescence after binding with breaks	Denatured DNA emit an orange-red fluorescence, normal DNA emits green
SCD/Halo test	To detect DNA breaks in lysed sperm nuclei	Agarose-embedded sperm are subjected to a denaturing solution to remove nuclear proteins	Assesses dispersion of DNA fragments after denaturation	Sperm with fragmented DNA do not produce the halo characteristic; halo of dispersed DNA loops are observed in sperm with non-fragmented DNA
SCGE/ Comet assay	To detect DNA single-strand breaks, ALS, and cross-linking	Gel electrophoresis	Electrophoretic assessment of DNA fragments	Comet tail size represents the amount of DNA fragments

ALS, alkali-labile sites; AO, acridine orange; CMA3, chromomycin A3; SCD, sperm chromatin dispersion; SCGE, single-cell gel electrophoresis, SCSA, sperm chromatin structure assay; TUNEL, terminal deoxynucleotidyl transferase nick-end labelling.

**Table 4:** Main non-enzymatic antioxidants used to treat male infertility.

Antioxidant	Dose	Effects on Sperm Parameters/Quality	References
CoQ10	200–300 mg/day	Improved sperm motility and TAC concentrations and decreased MDA levels.	[153,155,156]
Carnitines	25 mg/day	Improved sperm count, motility, and morphology.	[157,158,159]
Lycopene	20–25 mg/day	Increased seminal omega-3; improved sperm count, concentration, motility; and improved TAC; decreased seminal oxidative stress.	[154,160,161]
NAC	600 mg/day	Reduced apoptotic rate; improved sperm morphology, volume, motility, viscosity, TAC, DNA fragmentation, and protamine deficiency; decreased ROS production.	[162,163,164]
Melatonin	N/A	Sperm melatonin incubation was positively correlated with reduced DNA damage, MDA levels and higher sperm viability and motility.	[165,166,167,168,169]
Alpha-lipoic acid	600 mg/day	Improved sperm viability, motility, count, concentration, and TAC; decreased DNA damage and MDA levels.	[170,171,172,173]
Omega-3	1.5–2.0 g/day	Improved sperm volume, count, concentration, motility, and morphology; improved testis size, TAC, and reduced DNA fragmentation.	[174,175,176]

CoQ10, Q-10 coenzyme; MDA, malondialdehyde; ROS, reactive oxygen species; TAC, total antioxidant capacity.

**Table 5:** Some vitamins and minerals used as supplements to treat male infertility.

Vitamins/ Minerals	Dose	Main Conclusions	References
Vitamin C	1.0 g/day	Improved semen agglutination and sperm concentration, motility, and viability; positively associated with higher fertilization rates.	[182,185]
Vitamin E	100–600 mg/day	Decreased MDA levels and increased fertilization rates.	[186]
Vitamin B9	5 mg/day	Improved sperm count.	[187]
Zinc	200–500 mg/day	Improved sperm count, motility, and fertilization rates and reduced the incidence of anti-sperm antibodies; improved sperm chromatin integrity.	[188,189]
Selenium	200–1000 μg/day	Improved TAC and sperm count, concentration, motility, and morphology; positively associated with pregnancy and live birth.	[190,191,192,193,194]

MDA, malondialdehyde; TAC, total antioxidant capacity.

**Table 6:** Impact of positive lifestyle change on male fertility.

Factors	Findings	References
Dietary patterns	Healthy dietary patterns (Mediterranean and paleo diet) with low-fat and high non-dairy protein (i.e., fish and white meat) has an important influence on fertility. Dairy products rich in calcium and protein are beneficial. Diets with a low-glycemic load and high amounts of whole grains may benefit fecundity.	[31,32,33,40,209,210]
Oily sea fish, olive oil, and rapeseed oil intake	Fish and seafood are the main sources of omega-3 and fat-soluble vitamins A, D, E, and K; therefore, their incorporation into the diet may improve semen quality. Vegetable oils containing unsaturated acid residues, alpha-linolenic acid, vitamin E, and polyphenols can benefit fertility.	[13,37,211]
Vegetable, fruit, nut, and seed intake	Vegetables and fruits provide antioxidants, folic acid, fiber, and minerals associated with improved semen quality and fertility. Nuts and unroasted unsalted seeds provide fiber, tocopherols, phytosterols, polyphenols, and minerals that have a beneficial effect on sperm quality.	[35,36,37,196,212]
Whole-grain products in the diet	It is recommended that refined flour products be limited in the diet, with whole-grain products that are rich in fiber being more beneficial for fertility.	[36,213]
Physical exercise	Along with a healthy diet, regular exercise can improve BMI and fertility. It affects general health and well-being and protects against cardiovascular disease, type 2 diabetes, and psychological stress, among others.	[214,215,216,217]
Resistance training	Combined aerobic and resistance training, moderate-intensity continuous training, high-intensity continuous training, resistance training, and high-intensity interval training strategies improved semen quality parameters, seminal markers of oxidative stress, seminal markers of inflammation, as well as measures of body composition.	[218,219,220]
Sleep	Adequate sleep is crucial for general health and well-being. The relationship between sleep and reproductive hormones is bidirectional; reproductive hormones may modify sleep, and sleep disruption may alter the profile of reproductive hormone secretion. Multiple pathways exist by which sleep and circadian rhythms influence fertility. Additionally, good sleep can reduce mental stress.	[221,222,223,224]
Proactive stress reduction	Yoga and mindfulness training benefits fertility by reversing cellular dysfunctions in male reproductive organs and alleviates mental disturbances that influence reproduction.	[225,226,227,228]

BMI, body mass index.
